# QT Variability Index is Correlated with Autonomic Nerve Activity in Healthy Children

**DOI:** 10.1007/s00246-020-02399-8

**Published:** 2020-06-22

**Authors:** Hirofumi Kusuki, Yuka Tsuchiya, Yuri Mizutani, Miki Nishio, Shota Oikawa, Rina Nagata, Yumi Kiriyanagi, Kayo Horio, Arisa Kojima, Hidetoshi Uchida, Namiko Kojima, Kazuyoshi Saito, Tsuneaki Sadanaga, Tadayoshi Hata

**Affiliations:** 1 Graduate School of Health Sciences, Fujita Health University, Toyoake, Aichi Japan; 2 Laboratory of Clinical Medicine, Fujita Health University Hospital, Toyoake, Aichi Japan; 3 Department of Physiology, School of Medicine, Fujita Health University, Toyoake, Aichi Japan; 4 Department of Pediatrics, School of Medicine, Fujita Health University, Toyoake, Aichi Japan; 5 Department of Pediatrics, Meijyo Hospital, Nagoya, Aichi Japan; 6 Seigato Hospital, Kumamoto, Japan

**Keywords:** QT variability index, Heart rate variability, Prepubescent, LF/HF, HF/(LF + HF)

## Abstract

The QT variability index (QTVI), which measures the instability of myocardial repolarization, is usually calculated from a single electrocardiogram (ECG) recording and can be easily applied in children. It is well known that frequency analysis of heart rate variability (HRV) can detect autonomic balance, but it is not clear whether QTVI is correlated with autonomic tone. Therefore, we evaluated the association between QTVI and HRV to elucidate whether QTVI is correlated with autonomic nerve activity. Apparently, healthy 320 children aged 0–7 years who visited Fujita Health University Hospital for heart checkup examinations were included. The RR and QT intervals of 60 continuous heart beats were measured, and the QTVI was calculated using the formula of Berger et al. Frequency analysis of HRV, including the QTVI analysis region, was conducted for 2 min and the ratio of low-frequency (LF) components to high-frequency (HF) components (LF/HF) and HF/(LF + HF) ratio was calculated as indicators of autonomic nerve activity. Then, the correlations between QTVI and these parameters were assessed. QTVI showed a significant positive correlation with LF/HF ratio (*r* = 0.45, *p* < 0.001) and negative correlation with HF/(LF + HF) ratio (*r* = −0.429, *p* < 0.001). These correlations remained after adjustment for sex and age. QTVI, which is calculated from non-invasive ECG and can detect abnormal myocardial repolarization, is significantly correlated with frequency analysis of HRV parameters. QTVI reflects autonomic nerve balance in children.

## Introduction

Instability of myocardial repolarization detected by the QT interval variability indicates a substrate that can induce lethal arrhythmias [1]. Adult patients with myocardial dysfunction exhibit high QT interval variability following the preceding cardiac cycle, and such increased QT variability may be an ominous sign of cardiac death [2]. On the other hand, heart rate variability (HRV) calculated from the variations in the RR interval reflects autonomic nerve balance. Reduced HRV can predict the poor prognosis in patients with heart diseases [3, 4]. However, there were limited studies assessing the myocardial repolarization in children, and sufficient clinical applications have not been achieved.

The changes in QT interval variability are linked to autonomic and central nervous system [5]. The QT Variability Index (QTVI) is a non-invasive measure to assess repolarization liability that has been applied to a wide variety of subjects with cardiovascular disease [6]. We have used QTVI to evaluate the characteristics of myocardial repolarization in infants. This index changes with age in healthy children from infancy to school age [7, 8]. In healthy 1-month-old infants, the QTVI is negatively correlated with gestational age, which can serve as an index of the maturity of the cardiac autonomic nervous system and myocardial repolarization [9]. In the present study, we examined the correlation between QTVI and the power spectral analysis parameters of HRV, which are commonly used to measure autonomic nerve balance.

### Participants and Methods

Apparently healthy 320 children aged 0–7 years who visited Fujita Health University Hospital for heart checkup examinations between April 2012 and November 2015 were included. For children aged younger than 1 year, tricloryl syrup (0.7 mL/kg) was used for sedation to perform such diagnostic procedures. Informed consents were obtained from the children’s parents or guardians.

ECG was performed using a bio-polygraph recorder (MP-150; Biopac Systems Inc., CA, USA) with a sampling rate of 1000 Hz, and signals of the CM5 lead were recorded. All ECG recordings were obtained between 4 PM and 6 PM.

**Fig. 1:**
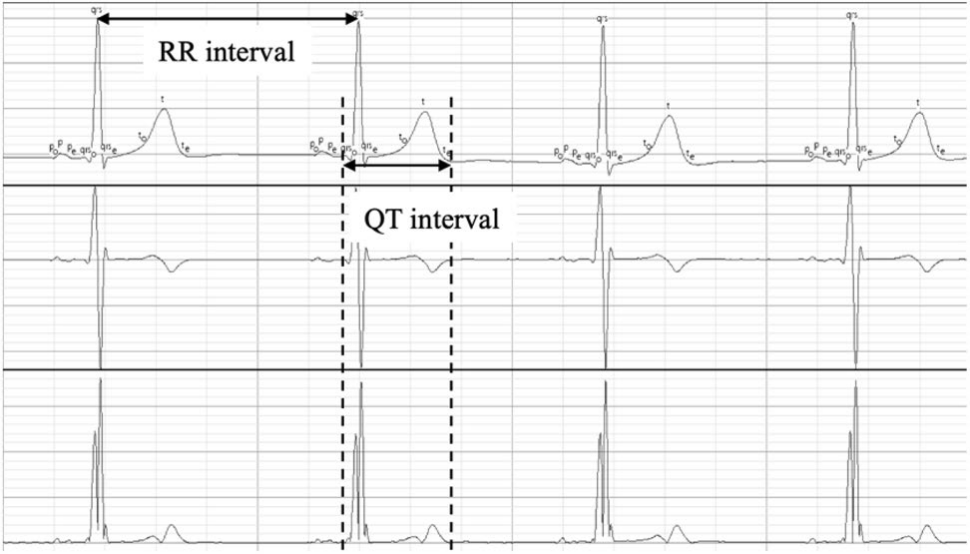
Measurement of RR interval and QT interval. The ECGs were recorded by the CM5 lead using a Biopac biological polygraph recording device. Q onset, T end, and preceding RR intervals were measured using first derivative (**b**) and absolute functions (**c**) from 60 consecutive beats with a stable baseline ECG

The RR interval was automatically measured using AcqKnowledge version 3.9 (Biopac Systems Inc., CA, USA) on the basis of the ECG recordings with stable baseline. The endpoint of the T wave was identified using the first-order differentiation processing method (Fig. 1). For 60 heart beats, we calculated the instantaneous heart rate, as well as the mean HR (HRm), mean QT interval (QTm), and variance (HRv and QTv). Thereafter, using the formula of Berger et al., we calculated the QTVI [QTVI = log_10_ (QTv/QTm^2^)/(HRv/HRm^2^)], which is an indicator of variability in the QT interval.

Meanwhile, to evaluate the autonomic nerve function, we performed power spectrum analysis of HRV during a short period of 2 min including the QTVI analysis region using AcqKnowledge version 3.9. In power spectrum analysis, we measured the frequency density of the low-frequency region (LF: 0.036–0.146 Hz) and the high-frequency region (HF: 0.146–0.390 Hz) according to the method shown in The Task Force of the European Society of Cardiology and the North American Society of Pacing and Electrophysiology [10]. Then, LF/HF and HF/(LF + HF) were calculated as indicators of autonomic nerve balance. Then, the correlations between QTVI and these parameters were assessed. The association of time domain HRV and QTVI was also assessed. In this study, standard deviation of NN intervals (SDNN) and root mean square of successive RR interval differences (rMSSD) were used. These values were expressed in original units or as the logarithm from (log_10_) to obtain normal distribution.

### Statistical Analysis

Statistical processing was performed using the JMP version 12.2.0 (SAS Institute Inc., Cary, NC, USA). The Wilcoxon signed rank test was used for Sex-specific comparison. Correlation between QTVI and age was assessed using logarithmic curve regression analysis. Correlations between QTVI and LF/HF or HF/(LF + HF) were assessed using Pearson’s linear regression analysis. Additionally, correlations between QTVI and SDNN or rMSSD were assessed using logarithmic curve regression analysis.

Multiple regression analysis was performed to assess whether QTVI was associated with HRV parameters independent of age and sex. A *p* value of < 0.05 was accepted as statistically significant.

## Results

### Participants’ Characteristics, ECG Parameters and HRV Parameters

Table 1 presents the characteristics of the participants. The median age was 3 years. Median QT interval was 318.6 ms with a median heart rate of 103.3 beats per minutes. The corrected QT intervals by Bazett’s and Fridericia’s formulas were 414.8 and 380.0 ms, respectively. None of the children had a corrected QT interval exceeding 440 ms. In the HRV parameters, median LF/HF ratio, reflecting sympathetic nerve activity, was 1.75, whereas median HF/(LF + HF), reflecting vagal nerve activity, was 0.36.

**Table 1:** Characteristics of the study population

Clinical characteristics	
Number	320
Male*I*Female	177/143
Age (months)	42.5 [16.0−64.0]
Age (years)	3.0 [1.0−5.0]
HRV parameters	
LF (ms^2^)	1716 [925−2893]
HF (ms^2^)	964 [443−2120]
LF/HF	1.75 [0.89−3.48]
HF/(LF + HF)	0.36 [0.22−0.53]
ECG parameters	
HR (bpm)	103.3 [91.9−115.1]
RR (ms)	583.4 [521.9−657.8]
QT (ms)	318.6 [294.4−336.5]
QTcB (ms)	414.8 [402.8−426.3]
QTcF (ms)	380.0 [364.5−392.1]
HRv (bpm^2^)	25.4 [13.5−39.5]
QTv (ms^2^)	15.8 [8.2−28.8]
log_10_HRVN	−2.63 [−2.95 to −2.39]
log_10_QTVN	−3.80 [−4.07 to −3.51]
QTVI	1.23 [−1.53 to −0.80]

Each value is expressed as median [interquartile range]. Comparisons between male and female were performed with the Wilcoxon signed rank test

*LF* low frequency density, *HF* high frequency density, *LF/HF* the ratio of low-frequency components to high-frequency components, *HF/(LF + HF)* the ratio of high-frequency components to low-frequency + high-frequency components, *HR* heart rate, *RR* RR interval, *QT* QT interval, *QTcB* corrected QT interval by Bazett’s formula, *QTcF* corrected QT interval by Fridericia’s formula, *HRVN* normalized HR variance, *QTVN* normalized QT variance, *QTVI* QT variability index, *β* standard regression coefficient, *SEM* standard error of the mean

### Gender Differences in QTVI and HRV Parameters

There were no gender differences for the QTVI, log_10_HRVN, and log_10_QTVN (Table 2).

**Table 2:** Gender difference in QTVI and HRV parameters

	Male	Female	*P*
QTVI	−1.23 [−1.52 to −0.77]	−1.25 [−1.54 to −0.86]	0.632
log_10_HRVN	−2.64 [−3.04 to −2.37]	−2.59 [−2.8754 to −2.41]	0.531
log_10_QTVN	−3.78 [−4.05 to −3.51]	−3.82 [−4.0854 to −3.52]	0.684

### Relationships Between QTVI and Age, Between LF/HF and Age

Figure 1 presents the relationship between QTVI and age and between LF/HF and age. Both indices decreased rapidly up to 12 months of age and slowly decreased thereafter.

### Relationship Between QTVI and LF/HF, Between QTVI and HF/(LF + HF)

**Fig. 2:**
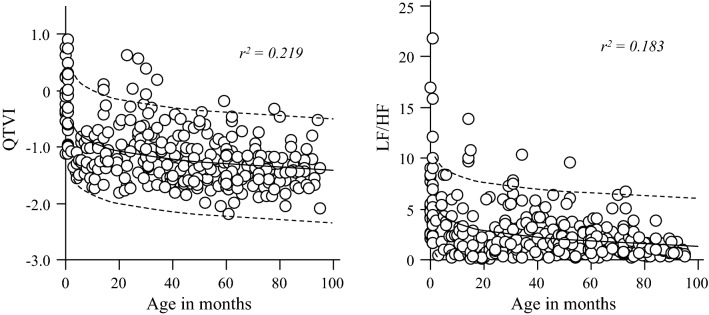
Relationships between QTVI or LF/HF and age in months. QTVI and LF/HF decreased rapidly until 12 months after birth, following which it gradually decreased during infancy and thereafter became constant when they reached preschool age

**Fig. 3:**
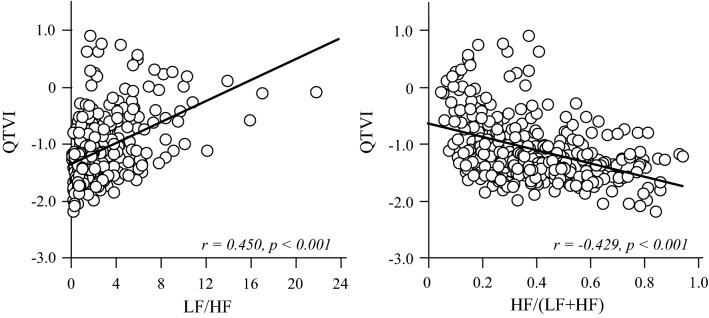
Relationships between QTVI and HRV parameters and QTVI and LF/HF show a positive correlation (*r* = 0.450, *p* < 0.001). QTVI and HF/(LF + HF) show a significant negative correlation (*r* = − 0.429, *p* < 0.001). *LF/HF* the ratio of low-frequency components to high-frequency components, *HF/(LF + HF)* the ratio of high-frequency components to low-frequency + high-frequency components

Figure 2 presents the relationship between QTVI and LF/HF and between QTVI and HF/(LF + HF). A significant positive correlation was observed between QTVI and LF/HF (*r* = 0.450, *p* < 0.001), whereas a significant negative correlation was observed between QTVI and HF/(LF + HF) (*r* = − 0.429, *p* < 0.001). A significant correlation between Log_10_HRVN and LF/HF, HF/(LF + HF) were observed (*r* = − 0.415, *p* < 0.001, *r* = 0.386, *p* < 0.001, respectively). Similar correlation between log_10_QTVN and LF/HF, HF/(LF + HF) were observed (*r* = 0.144, *p* = 0.010, *r* = −0.151, *p* = 0.007, respectively), but the correlations were weak (Fig. 3).

### Relationship Between QTVI and SDNN, Between QTVI and rMSSD

A significant correlation between QTVI and log_10_SDNN and log_10_rMSSD was observed (*r* = − 0.784, *p* < 0.001, *r* = 0.756, *p* < 0.001, respectively).

### Multivariate Analysis

Table 3 shows multiple regression analysis. QTVI was significantly correlated with LF/HF and HF/(LF + HF) after adjustment for age and sex.

**Table 3:** The multiple regression analysis with QTVI as age, sex, and HRV parameters

Independent variable	*P*	SEM	*P*
Age (years)	−0.291	0.044	< 0.001
Sex	0.019	0.054	0.723
LF/HF	0.711	0.113	< 0.001
Age (years)	−0.313	0.043	< 0.001
Sex	0.038	0.054	0.486
HF/(LF + HF)	−0.400	0.062	< 0.001

## Discussion

The present study demonstrated that the QTVI is correlated with autonomic nerve activity in prepubescent healthy children aged 0–7 years. Namely, QTVI showed a significant positive correlation with LF/HF ratio reflecting sympathetic nerve activity and negative correlation with HF/(LF +HF) ratio, reflecting vagal nerve activity. QTVI showed a significant negative correlation with SDNN reflecting the sympatho-vagal nervous balance and negative correlation with rMSSD, reflecting vagal nerve activity.

Berger et al. [2] proposed that temporal variations in the QT interval—instability in myocardial repolarization—could serve as an electrophysiological indicator. QT interval is affected by the heart rate; larger HRV could induce larger QT interval variability, whereas smaller HRV could induce smaller QT interval variability. Either overestimation or underestimation of QT variability might occur without considering the degree of HRV. Therefore, the authors proposed the formula: QTVI = log_10_ (QTv/QTm^2^)/(HRv/HRm^2^)] taking into account the effects of HRV. In fact, this formula has been evaluated in clinical studies and proved to be useful in distinguishing patients with high risk for sudden cardiac death in hypertrophic cardiomyopathy and patients with a history of ventricular fibrillation [11]. Although all studies did not always present data of the numerator (QTVN: QT variance/QTmean^2^) and denominator (HRVN: HR variance/HRmean^2^), increased QTVN rather than decreased HRVN might usually be the cause of QTVI to increase. Dobson suggested that evaluating both QTVN and HRVN is important in assessing the pathophysiology of QTVI. [6] As previously shown, HRVN changes with age; however, QTVN was not affected by age and remained stable without sex-related differences in children [7]. Thus, increase in QTVI might not necessarily indicate increase in QTVN, but could be due to decrease in HRVN. Actually, our analysis revealed a significant relationship between LF/HF and HF/(LF + HF) with log_10_HRVN; however, only weak relationship was observed with log_10_QTVN. Therefore, it might be possible that increased sympathetic nerve activity reduces RR interval variability but has no marked effect on the QT interval variability. Schmidt M and colleagues showed that QTV was increased in rapid eye movement sleep, reflective of high sympathetic drive and predicts death from cardiovascular disease [12]. However, HRV was not assessed in the paper. Likewise, augmented vagal nerve activity increases RR interval variability and could decrease QTVI, but its effect on QTVN is not clear. Further studies are required to evaluate the relationship between autonomic nerve balance and QTVN.

### Potential Clinical Implication

Sudden Infant Death Syndrome (SIDS) is one of the leading causes of death in infants. Although the exact mechanisms contributing SIDS have not been fully elucidated until now, it is estimated that dysfunction of the autonomic nervous system regulation either respiratory or cardiovascular systems might play a part. The advantage of this study is that QTVI, which reflects myocardial repolarization variability, calculated from short-term ECG recording, can be used to assess the degree of autonomic tone. Thus, QTVI could be potentially used for predicting lethal arrhythmias, including SIDS. This should be explored in the future studies.

## Limitations

This study has some limitations. First, this was a single-center study with a limited number of patients.

Second, we collected only one data in each subject. Thus, reproducibility of the parameters could not be assessed. Third, it may be true that sedatives affect the autonomic nervous activity and should be avoided to assess the autonomic balance, but in fact, it was impossible to ask the infant to rest on supine position for more than 2 min. However, the amount of tricloryl syrup (0.7 mg/kg) used in this study is small so that we think effects of this medication on autonomic nervous activity is limited. Finally, we included only healthy infants, therefore it is unclear whether our results could be applied to infants with heart disease including hereditary channelopathies.

## Conclusion

QTVI, which is calculated from non-invasive ECG and can detect abnormal myocardial repolarization instability, is significantly correlated with power spectral HRV parameters. QTVI reflects autonomic nerve balance in children.
